# FreeSurfer-based amygdala subfield volumetry in long COVID and ME/CFS: clinical and immunological correlations

**DOI:** 10.3389/fneur.2026.1913553

**Published:** 2026-09-16

**Authors:** Yukio Kimura, Wakiro Sato, Yoko Shigemoto, Risa Kagaya, Akihiko Hayakawa, Yunosuke Kumazawa, Isu Shin, Keiko Amano, Takashi Yamamura, Noriko Sato

**Affiliations:** 1 Department of Radiology, National Center Hospital of Neurology and Psychiatry, Kodaira, Tokyo, Japan; 2 Department of Immunology, National Institute of Neuroscience, National Center of Neurology and Psychiatry, Kodaira, Tokyo, Japan; 3 Sekimachi Medical Clinic, Nerima, Tokyo, Japan; 4 Seifuso Hospital, Niiza, Saitama, Japan

**Keywords:** amygdala, FreeSurfer, long COVID, ME/CFS, myalgic encephalomyelitis/chronic fatigue syndrome

## Abstract

**Background:**

Long COVID and myalgic encephalomyelitis/chronic fatigue syndrome (ME/CFS) share debilitating symptoms and have been associated with limbic-system dysfunction. We investigated amygdala subfield volumes to identify disease-specific neuroanatomical features and their clinical and immunological correlates.

**Methods:**

We prospectively examined 27 patients with long COVID, 38 patients with ME/CFS, and 47 healthy controls (HCs). Amygdala subfields were segmented using high-resolution 3-T MRI and FreeSurfer software. Group comparisons were performed using analysis of covariance, and partial correlations with clinical and immunological indices, including autoantibodies, plasmablasts, regulatory T cells (Tregs), and Eomesodermin-positive helper T cells (Eomes+ Th cells), were assessed.

**Results:**

Patients with long COVID had nominally larger right cortical nucleus volumes than HCs (raw *p* = 0.040; Bonferroni-adjusted *p* = 0.119), whereas patients with ME/CFS had a nominally larger left paralaminar nucleus (raw *p* = 0.048; Bonferroni-adjusted *p* = 0.145). In long COVID, G-protein-coupled receptor (GPCR) autoantibodies were nominally negatively associated with right medial nucleus volume. In ME/CFS, performance status was nominally positively associated with the right basal nucleus, right paralaminar nucleus, and right whole amygdala, and plasmablasts were nominally positively associated with the right corticoamygdaloid transition area. No group difference or partial correlation remained significant after correction for multiple testing.

**Conclusion:**

Although no finding survived correction for multiple testing, the exploratory results suggest that amygdala-related structural variation may be relevant to both long COVID and ME/CFS. The nominal, uncorrected within-group patterns centered on olfactory-related amygdala nuclei and GPCR autoantibodies in long COVID, and on amygdala structure, functional severity, and plasmablasts in ME/CFS; however, these patterns do not support a conclusion of between-group differences. These observations are hypothesis-generating and do not establish disease-specific mechanisms. Prespecified validation in larger longitudinal and independent cohorts is required.

## Introduction

Since the onset of the COVID-19 pandemic, many individuals worldwide have experienced persistent symptoms after SARS-CoV-2 infection, a condition collectively termed “long COVID.” A large-scale population-based study reported that approximately 13% of individuals experience persistent somatic symptoms attributable to COVID-19 (1, 2). Long COVID shares considerable clinical overlap with myalgic encephalomyelitis/chronic fatigue syndrome (ME/CFS). More than half of patients with long COVID reportedly meet the diagnostic criteria for ME/CFS, suggesting possible shared pathophysiological features between these conditions (3, 4).

The limbic system, which is involved in emotional and autonomic regulation, has been a major focus of neuroimaging research in these populations (5, 6). Although a recent high-resolution 7-Tesla MRI study reported subfield-level alterations in the hippocampus of patients with long COVID and ME/CFS (7), the amygdala—a key structure involved in stress responses and neuroendocrine regulation—has not yet been investigated at this level of anatomical detail (8). Subfield-level analysis may help identify localized abnormalities that are obscured by whole-structure volumetric measurements (9).

Our research group has focused on the neuroimmune axis in ME/CFS by combining advanced neuroimaging with detailed immunological assessments. Specifically, we have shown that autoantibodies against β-adrenergic and muscarinic receptors are associated with altered structural brain networks and microstructural brain changes detected using free-water-corrected diffusion tensor imaging (FW-DTI) (10, 11). Although several immunological abnormalities have been reported in long COVID (12, 13), few studies have integrated detailed immunological profiling with quantitative neuroimaging findings. Therefore, integrating amygdala subfield morphometry with such profiling may help clarify the neuroanatomical and pathophysiological relationship between long COVID and ME/CFS.

In the present study, we used automated segmentation to compare amygdala subfield volumes among patients with long COVID, patients with ME/CFS, and healthy controls. We also examined associations between these neuroanatomical measures and selected clinical and immunological parameters, including performance status (PS), autoantibody titers, and immune-cell profiles, within each patient group. The objective of this study was to compare amygdala subfield volumes among the three groups and to explore within-group associations between these volumes and selected clinical and immunological variables, thereby evaluating potentially shared and differing amygdala-related structural and clinical/immunological patterns in long COVID and ME/CFS.

## Materials and methods

### Participants

This prospective observational study was approved by the Ethics Committee of the National Center of Neurology and Psychiatry (NCNP), and all participants provided written informed consent. We prospectively evaluated 31 consecutive patients with long COVID and 70 consecutive patients with ME/CFS who underwent brain MRI at our institute between July 2020 and December 2024.

The inclusion criteria for the long COVID group were as follows: (1) fulfillment of the WHO clinical case definition of post-COVID-19 condition (14); (2) completion of clinical and laboratory evaluations, including assessment of the performance status (PS) score, a symptom-severity rating based on activities of daily living on a 0–9 scale, with higher scores indicating greater illness severity (15); measurement of autoantibodies against the β1-adrenergic receptor (β1 AdR-Ab), β2-adrenergic receptor (β2 AdR-Ab), M3 muscarinic acetylcholine receptor (M3 AchR-Ab), and M4 muscarinic acetylcholine receptor (M4 AchR-Ab) using CellTrend; and assessment of immune-cell parameters, including plasmablasts (PBs; % of B cells), regulatory T cells (Tregs; defined as CD3+CD4+CD127−CD25high cells; % of CD4+ T cells), and Eomesodermin-positive helper T cells (Eomes+ Th cells; % of CD4+ T cells) (16, 17); and (3) an interval of <30 days between MRI and these clinical/laboratory evaluations. The inclusion criteria for the ME/CFS group were as follows: (1) fulfillment of the Canadian Consensus Criteria for ME/CFS (18); (2) completion of the same clinical and laboratory evaluations as those described for the long COVID group; (3) an interval of <30 days between MRI and these evaluations; and (4) no history of COVID-19 infection. Patients with significant motion artifacts or abnormal MRI findings, such as tumor, demyelinating lesions, hemorrhage, or infarction, were excluded based on consensus readings by two board-certified neuroradiologists (Y. K. and R. K.). Of the 31 patients with long COVID assessed for eligibility, four were excluded because of incomplete immunological data (*n* = 2) or an interval of ≥30 days between MRI and the clinical/laboratory evaluations (*n* = 2). Of the 70 patients with ME/CFS assessed for eligibility, 32 were excluded because of incomplete immunological data (*n* = 12), an interval of ≥30 days (*n* = 11), unsuccessful FreeSurfer segmentation (*n* = 2), or incomplete clinical data (*n* = 7). After these exclusions, the final cohort included 27 patients with long COVID (mean ± SD age, 41.0 ± 10.2 years; 16 females) and 38 patients with ME/CFS (mean ± SD age, 41.8 ± 8.8 years; 22 females). As a comparison group, 47 age- and sex-matched healthy controls (HCs; mean ± SD age, 41.1 ± 9.9 years; 27 females) with no history of neuropsychiatric or systemic disease were included. None of the HCs had a history of SARS-CoV-2 infection, and their MRI data were acquired using the same scanner and imaging protocol as those used for the patient groups. Participant selection is summarized in Supplementary Figure S1.

### Clinical and immunological assessments

The PS score was recorded on a 0–9 activities-of-daily-living scale, with higher scores indicating greater illness severity (15). Serum IgG autoantibodies against the β1-adrenergic receptor, β2-adrenergic receptor, M3 muscarinic acetylcholine receptor, and M4 muscarinic acetylcholine receptor were quantified by enzyme-linked immunosorbent assay (ELISA) at CellTrend GmbH (Luckenwalde, Germany), as previously described (19). The β1- and β2-adrenergic receptors and the M3 and M4 muscarinic acetylcholine receptors are all members of the G-protein-coupled receptor (GPCR) family. Recombinant proteins corresponding to these four GPCRs, expressed in Chinese hamster ovary cells, were used as antigens. Clinical and immunological assessments were completed within 30 days of MRI.

For immune-cell profiling, peripheral blood mononuclear cells were isolated from heparinized blood by Ficoll-Paque Plus density-gradient centrifugation. Cells were stained with fluorescence-conjugated monoclonal antibodies and analyzed on a FACS Canto II or FACS Aria II flow cytometer (BD Biosciences) according to previously reported methods (16, 17, 20). Plasmablasts were defined as CD19+CD27+CD180−CD38high cells and expressed as a percentage of B cells; regulatory T cells were defined as CD3+CD4+CD127−CD25high cells and expressed as a percentage of CD4+ T cells; and Eomesodermin-positive helper T cells were defined as CD3+CD4+ helper T cells with intracellular Eomesodermin expression and expressed as a percentage of CD4+ T cells. Sample handling and flow-cytometric analysis were completed on the day of blood collection.

### Image acquisition and data preprocessing

All participants underwent MRI on a 3.0-T system (Achieva; Philips Medical Systems, Best, The Netherlands) using a 32-channel head coil. All scans were acquired using the same scanner, head coil, and imaging protocol across all groups, including the HCs. Three-dimensional sagittal T1-weighted magnetization-prepared rapid gradient-echo (MPRAGE) images were obtained using the following parameters: repetition time/echo time/inversion time, 7.2/3.5/1100 ms; flip angle, 10°; section thickness, 0.6 mm; number of slices, 300; matrix, 384 × 384; field of view, 261 × 261 mm; and number of excitations, 1. The acquisition time for the MPRAGE sequence was approximately 4 min.

All T1-weighted structural images were processed using the automated recon-all pipeline in FreeSurfer version 8.1.0.^1^ Bilateral amygdala volumes were segmented using the FreeSurfer 8.1.0 amygdala module (Figure 1). This process automatically defined nine bilateral subfields: the lateral, basal, accessory basal, central, medial, cortical, and paralaminar nuclei, as well as the anterior amygdaloid area and corticoamygdaloid transition. Whole-amygdala volume was also calculated. Each segmentation was manually inspected by two board-certified neuroradiologists to confirm anatomical accuracy and exclude processing artifacts. Estimated total intracranial volume (eTIV) was derived using FreeSurfer’s affine scaling method and included as a covariate in the statistical analysis.

**Figure 1:**
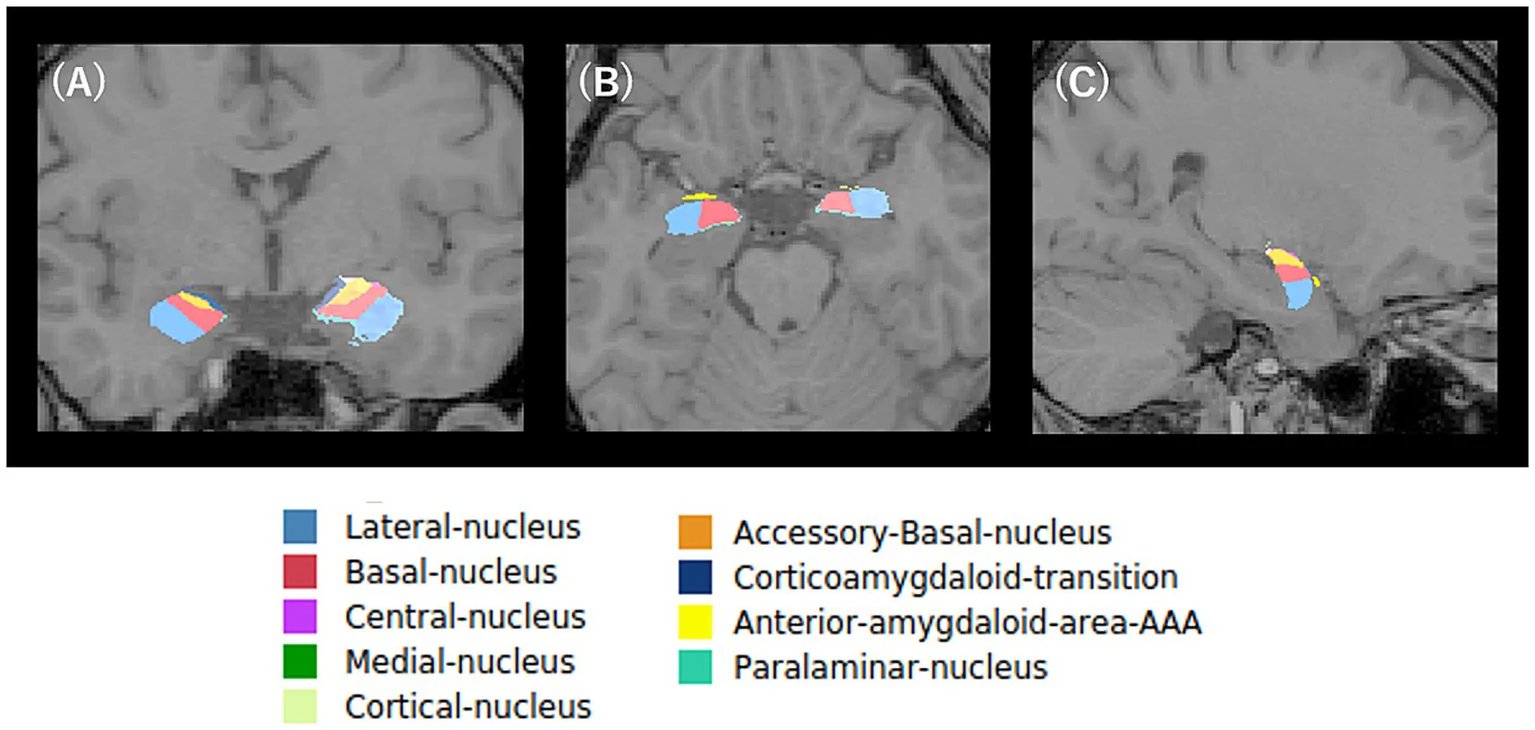
Automated segmentation of amygdala subfields. Representative T1-weighted structural images showing automated FreeSurfer-based segmentation of the amygdala subfields in the **(A)** coronal, **(B)** axial, and **(C)** sagittal planes.

### Statistical analysis

Continuous demographic and clinical variables were compared among the three groups using one-way analysis of variance (ANOVA) or Kruskal–Wallis tests and between the two patient groups using Student’s *t*-tests or Mann–Whitney *U* tests, as appropriate. Categorical variables, such as sex, were compared using chi-square tests. Amygdala subfield volumes were compared among the three groups using general linear models, with group and sex included as fixed factors and age and eTIV as covariates. The family of volumetric hypotheses comprised the 20 bilateral subfield and whole-amygdala outcomes; BH-FDR correction was applied across the 20 omnibus group-effect *p*-values. Pairwise group comparisons were performed within the analysis-of-covariance framework using estimated marginal means and were Bonferroni-corrected within each outcome. Omnibus *F* statistics, degrees of freedom, raw *p*-values, BH-FDR *q*-values, partial *η*^2^, adjusted pairwise mean differences, standard errors, Bonferroni-adjusted *p*-values, and 95% confidence intervals are reported in Supplementary Table S1. Associations between amygdala subfield volumes and clinical and immunological variables, including disease duration, PS, immune-cell parameters, and autoantibody titers, were evaluated separately in the long COVID and ME/CFS groups using partial Spearman rank correlations adjusted for age, sex, and eTIV. This rank-based method was selected because PS is an ordinal measure and disease duration and several immunological variables showed skewed distributions. Rank transformations were applied to the clinical, immunological, volumetric, and covariate variables before partial-correlation analysis. Nine clinical/immunological variables were tested against 20 amygdala outcomes, yielding 180 correlations per patient group; BH-FDR correction was applied separately within each group. An FDR-adjusted *q* < 0.05 was considered statistically significant, whereas associations with raw *p* < 0.05 were treated as nominal exploratory findings. Complete correlation results are provided in Supplementary Tables S2, S3. Tests were two-sided, and missing data were handled by listwise deletion within each analysis.

Model assumptions were evaluated using residual Q–Q plots, Shapiro–Wilk tests, Levene tests, plots of standardized residuals against fitted values, studentized residuals, and Cook distances. Linearity of covariate effects was assessed visually from residual-versus-fitted plots. Homogeneity of regression slopes was examined by adding group-by-age and group-by-eTIV interaction terms to the models.

## Results

### Demographics and clinical characteristics

The demographic and clinical characteristics of all participants are summarized in Table 1. Age and sex did not differ significantly among the three groups. Disease duration was significantly shorter in the long COVID group (mean ± SD, 0.94 ± 0.70 years; median, 0.58 years; interquartile range, 0.38–1.54 years; range, 0.33–3.00 years) than in the ME/CFS group (mean ± SD, 7.45 ± 7.85 years; median, 5.0 years; interquartile range, 1.0–10.0 years; range, 0.5–34 years; *p* < 0.001). Among the immunological parameters, the proportion of PBs was significantly higher in the long COVID group than in the ME/CFS group (*p* = 0.028). No other immune-cell parameters or autoantibody titers differed significantly between the two patient groups.

**Table 1:** Demographic and clinical characteristics of participants.

Variable	HC (*n* = 47)	Long COVID (*n* = 27)	ME/CFS (*n* = 38)	*p*-value^*^
Age, years	41.1 ± 9.9	41.0 ± 10.2	41.8 ± 8.8	0.790
Sex, male/female	20/27	11/16	16/22	1.00
Clinical indices				
Disease duration, years	–	0.94 ± 0.70	7.4 ± 7.9	< 0.001 *
PS score, range: 0–9	–	6.4 ± 1.3	6.0 ± 1.8	0.830
Immunological indices				
*Autoantibodies*				
β1 AdR-Ab, U/mL	–	19.9 ± 16.5	17.1 ± 16.2	0.480
β2 AdR-Ab, U/mL	–	18.4 ± 14.7	19.9 ± 18.2	0.760
M3 AchR-Ab, U/mL	–	13.5 ± 14.0	13.6 ± 15.3	0.960
M4 AchR-Ab, U/mL	–	15.4 ± 10.9	11.5 ± 9.7	0.160
*Immune-cell parameters*				
PB, %	–	3.9 ± 3.8	2.0 ± 1.6	0.028 *
Treg, %	–	5.3 ± 1.7	5.6 ± 2.0	0.740
Eomes+ Th, %	–	13.9 ± 12.0	11.2 ± 8.8	0.320

Data are presented as the mean ± standard deviation or number, as appropriate. *p*-values were calculated using one-way analysis of variance or the Kruskal–Wallis test for comparisons among the three groups, Student’s *t*-test or the Mann–Whitney *U* test for comparisons between the two patient groups, and the chi-square test for categorical variables, as appropriate. ^*^p < 0.05. AChR-Ab, acetylcholine receptor antibody; AdR-Ab, adrenergic receptor antibody; Eomes+ Th, Eomesodermin-positive helper T cells; HC, healthy controls; ME/CFS, myalgic encephalomyelitis/chronic fatigue syndrome; PB, plasmablasts; PS, performance status; Treg, regulatory T cells.

### Group comparisons of amygdala subfield volumes

Amygdala subfield volumes and eTIV are summarized in Table 2 as unadjusted means ± SDs. eTIV did not differ significantly among the three groups. None of the 20 omnibus group effects was significant before or after BH-FDR correction. The smallest raw omnibus *p*-value was observed for the right cortical nucleus [*F*(2,106) = 2.557, raw *p* = 0.082, partial *η*^2^ = 0.046, *q* = 0.476]. In exploratory uncorrected pairwise comparisons, patients with long COVID had a nominally larger right cortical nucleus than HCs (adjusted mean difference, 2.275 mm^3^; raw *p* = 0.040), and patients with ME/CFS had a nominally larger left paralaminar nucleus than HCs (adjusted mean difference, 1.880 mm^3^; raw *p* = 0.048). Neither comparison remained significant after Bonferroni correction (adjusted *p* = 0.119; Bonferroni-adjusted 95% CI, −0.382 to 4.933, and adjusted *p* = 0.145; Bonferroni-adjusted 95% CI, −0.410 to 4.170, respectively), and no direct long COVID–ME/CFS comparison was significant. These findings are therefore presented as candidate subfield-level patterns rather than confirmed group differences. Full model results are provided in Supplementary Table S1.

**Table 2:** MRI-derived volumetric measures of eTIV and amygdala subfields.

Amygdala subfield volumes (mm^3^)	HC (*n* = 47)	Long COVID (*n* = 27)	ME/CFS (*n* = 38)	Raw pairwise *p*-value		
				Long COVID vs. HC	ME/CFS vs. HC	Long COVID vs. ME/CFS
Left lateral nucleus	695.3 ± 73.6	695.0 ± 75.0	700.1 ± 69.6	0.408	0.133	0.596
Left basal nucleus	450.5 ± 49.7	447.8 ± 48.2	454.1 ± 53.6	0.506	0.072	0.341
Left accessory basal nucleus	276.5 ± 29.9	272.1 ± 29.7	278.4 ± 36.2	0.817	0.071	0.168
Left anterior amygdaloid area	57.9 ± 7.9	57.7 ± 9.2	58.5 ± 7.6	0.539	0.169	0.531
Left central nucleus	52.5 ± 11.4	51.5 ± 13.5	50.6 ± 10.8	0.988	0.814	0.824
Left medial nucleus	27.7 ± 7.2	27.5 ± 8.9	25.7 ± 7.4	0.768	0.512	0.388
Left cortical nucleus	28.5 ± 4.4	28.1 ± 5.3	28.7 ± 5.5	0.751	0.231	0.451
Left corticoamygdaloid transition area	174.3 ± 19.3	171.8 ± 17.3	173.4 ± 22.1	0.881	0.355	0.501
Left paralaminar nucleus	46.4 ± 5.4	46.7 ± 5.1	47.2 ± 6.3	0.288	0.048^*^	0.466
Left whole amygdala	1809.7 ± 178.7	1798.1 ± 179.4	1816.7 ± 188.9	0.462	0.075	0.385
Right lateral nucleus	707.2 ± 65.2	703.0 ± 86.8	714.3 ± 83.5	0.571	0.104	0.372
Right basal nucleus	467.6 ± 45.5	469.8 ± 58.0	466.9 ± 57.3	0.307	0.304	0.936
Right accessory basal nucleus	290.0 ± 29.2	293.1 ± 35.0	293.0 ± 40.1	0.175	0.081	0.816
Right anterior amygdaloid area	63.9 ± 8.4	64.0 ± 9.9	65.5 ± 8.4	0.493	0.080	0.372
Right central nucleus	55.9 ± 9.7	56.6 ± 12.5	54.4 ± 11.9	0.443	0.931	0.413
Right medial nucleus	28.7 ± 7.4	29.1 ± 7.7	28.3 ± 8.0	0.404	0.620	0.712
Right cortical nucleus	30.2 ± 4.8	31.7 ± 5.6	30.9 ± 5.6	0.040^*^	0.097	0.591
Right corticoamygdaloid transition area	176.9 ± 18.6	178.4 ± 16.5	178.7 ± 22.8	0.219	0.081	0.720
Right paralaminar nucleus	46.2 ± 5.1	46.7 ± 6.4	45.9 ± 5.1	0.175	0.426	0.540
Right whole amygdala	1866.6 ± 168.1	1872.5 ± 211.4	1878.0 ± 214.7	0.247	0.089	0.700
eTIV, mm^3^	1,438,477 ± 138,507	1,446,145 ± 131,049	1,452,662 ± 130,551	0.890		

Data are presented as the mean ± standard deviation. Pairwise raw *p*-values for amygdala subfield volumes were calculated within an analysis-of-covariance framework adjusted for age, sex, and eTIV. Bonferroni-adjusted *p*-values and 95% confidence intervals are provided in Supplementary Table S1. ^*^Raw *p* < 0.05; no pairwise comparison remained significant after Bonferroni correction. eTIV, estimated total intracranial volume; HC, healthy controls; ME/CFS, myalgic encephalomyelitis/chronic fatigue syndrome.

Model diagnostics showed no substantial violations that materially altered interpretation. Residual Q–Q plots were generally acceptable, although Shapiro–Wilk tests indicated right-tail departures for the bilateral anterior amygdaloid area and paralaminar nucleus residuals. Levene testing was nominally significant only for the left central nucleus (*p* = 0.036); residual-versus-fitted plots showed no clear nonlinear or funnel-shaped pattern. Maximum Cook distances ranged from 0.07 to 0.17, indicating no unduly influential observations. Group-by-age and group-by-eTIV interactions were nonsignificant for all 20 outcomes (minimum *p* = 0.052 and 0.056, respectively), supporting homogeneity of regression slopes.

### Correlation analyses with clinical and immunological variables

Partial Spearman rank-correlation analyses adjusted for age, sex, and eTIV identified three of 180 nominal associations in the long COVID group and four of 180 in the ME/CFS group (Table 3); none survived BH-FDR correction. In the long COVID group, β1-adrenergic receptor autoantibodies (*ρ* = −0.614, raw *p* = 0.001, *q* = 0.180), β2-adrenergic receptor autoantibodies (*ρ* = −0.557, raw *p* = 0.005, *q* = 0.450), and M3 muscarinic acetylcholine receptor autoantibodies (*ρ* = −0.464, raw *p* = 0.022, *q* = 0.992) were nominally negatively associated with right medial nucleus volume. In the ME/CFS group, PS was nominally positively associated with the right basal nucleus (*ρ* = 0.392, raw *p* = 0.020, *q* = 0.957), right paralaminar nucleus (*ρ* = 0.407, raw *p* = 0.015, *q* = 0.957), and right whole amygdala (*ρ* = 0.350, raw *p* = 0.039, *q* = 0.957). Plasmablast percentage was nominally positively associated with the right corticoamygdaloid transition area (*ρ* = 0.460, raw *p* = 0.005, *q* = 0.900). Complete results for all 180 correlations in each patient group are provided in Supplementary Tables S2, S3.

**Table 3:** Nominal partial Spearman rank correlations between amygdala subfield volumes and clinical/immunological variables (raw *p* < 0.05).

Group	Variable	Amygdala subfield	Partial Spearman *ρ*	Raw *p*/BH-FDR *q*
Long COVID	Immunological			
	β1 AdR-Ab	Right medial nucleus	−0.614	0.001/0.180
	β2 AdR-Ab	Right medial nucleus	−0.557	0.005/0.450
	M3 AchR-Ab	Right medial nucleus	−0.464	0.022/0.992
ME/CFS	Clinical			
	PS	Right basal nucleus	0.392	0.020/0.957
	PS	Right paralaminar nucleus	0.407	0.015/0.957
	PS	Right whole amygdala	0.350	0.039/0.957
	Immunological			
	PB	Right corticoamygdaloid transition area	0.460	0.005/0.900

Partial Spearman rank correlations were adjusted for age, sex, and estimated total intracranial volume. *ρ* represents the partial Spearman rank-correlation coefficient. Raw *p*-values and BH-FDR *q*-values are shown. No association remained significant after BH-FDR correction. AChR-Ab, acetylcholine receptor antibody; AdR-Ab, adrenergic receptor antibody; ME/CFS, myalgic encephalomyelitis/chronic fatigue syndrome; PB, plasmablasts; PS, performance status.

## Discussion

In the present study, we performed nucleus-level amygdala subfield volumetry in patients with long COVID and ME/CFS and examined associations between amygdala subfield volumes and clinical and immunological parameters using the same protocol. In exploratory uncorrected pairwise comparisons, patients with long COVID showed a nominally larger right cortical nucleus than healthy controls, whereas patients with ME/CFS showed a nominally larger left paralaminar nucleus. However, neither comparison remained significant after Bonferroni correction, and no omnibus group effect or partial correlation remained significant after correction for multiple testing. Taken together, these exploratory findings raise the possibility that amygdala-related structural variation may be relevant to both conditions. The nominal, uncorrected within-group patterns involved olfactory-related amygdala nuclei and GPCR autoantibody profiles in long COVID, and amygdala structure, PS, and plasmablast percentage in ME/CFS; however, these patterns do not support a conclusion of between-group differences. These hypotheses do not establish disease specificity or causality and require prespecified evaluation in larger independent cohorts.

The nucleus-specific nature of these findings is clinically and biologically relevant. Previous ME/CFS neuroimaging studies have mainly used voxel-based morphometry or whole-structure volumetric analyses (5, 6), whereas recent ultra-high-field MRI work has focused primarily on hippocampal subfields (7). Because the amygdala comprises multiple nuclei with distinct connectivity and functions, whole-amygdala measurements may obscure localized disease-related alterations (8, 9, 21). Therefore, the present study extends prior work by linking amygdala nucleus-level structural changes to peripheral immune profiles.

In patients with long COVID, β1- and β2-adrenergic receptor autoantibodies and M3 muscarinic acetylcholine receptor autoantibodies were nominally negatively associated with right medial nucleus volume. The medial nucleus is a primary olfactory-related amygdala region. β2-Adrenergic and muscarinic receptors belong to the GPCR family, and functional autoantibodies against these receptors have been described in both long COVID and ME/CFS, particularly in patients with fatigue and dysautonomia (19, 22–24). However, none of the present associations survived FDR correction, and these cross-sectional correlations do not establish a causal or mechanistic link between peripheral autoantibodies and amygdala structure.

In our cohort, the long COVID group exhibited a nominally larger right cortical nucleus than healthy controls. Both the medial and cortical nuclei are primary olfactory-related amygdala regions. Together, these findings may identify an olfactory–limbic candidate pattern in long COVID. However, T1-weighted volumetry alone cannot determine whether volume variation reflects inflammation, edema, vascular or hydration effects, neuroplasticity, interindividual variation, or segmentation variability. Because disease duration was markedly shorter in long COVID than in ME/CFS, an illness-stage explanation is possible, but this remains a provisional working hypothesis that requires longitudinal evaluation.

In patients with ME/CFS, PS scores were nominally positively associated with the right basal nucleus, right paralaminar nucleus, and right whole-amygdala volume, suggesting a possible relationship between functional severity and amygdala structural variation. The strongest association was observed in the basal nucleus, a region connected with the hippocampus, medial prefrontal cortex, and insula that is involved in contextual emotional learning and interoceptive integration (25, 26). However, PS is a broad functional measure; detailed fatigue, post-exertional, autonomic, cognitive, olfactory, and psychiatric domains were not assessed, and these nominal associations do not demonstrate severity-driven remodeling.

In the ME/CFS group, plasmablast percentage was nominally positively associated with right corticoamygdaloid transition area volume. Plasmablasts reflect active B-cell differentiation and recent or ongoing antigen-driven humoral responses (27). Previous reports of B-cell receptor repertoire skewing and immune abnormalities in ME/CFS support further investigation of this candidate association (20, 28). Although this association did not survive FDR correction, its localization to the corticoamygdaloid transition area, an olfactory–limbic interface, may warrant further investigation. Previous reports of structural changes in olfactory-related cortical and limbic regions after SARS-CoV-2 infection (29) may provide broader neuroanatomical context for future studies.

The nominal within-group associations do not demonstrate statistically different disease-specific patterns. Formal group-by-biomarker interaction testing or direct comparison of correlation coefficients would be required, and the current study was not powered for these analyses. Disease duration also differed markedly between groups, with medians of 0.58 years in long COVID and 5.0 years in ME/CFS. Diagnostic group and illness duration are therefore strongly confounded, and the observed patterns could reflect diagnosis, illness stage, cohort composition, or other unmeasured factors. Longitudinal studies with overlapping duration ranges are needed to separate these possibilities. Although these findings may identify candidate structural correlates, their potential value as clinical biomarkers remains uncertain because diagnostic accuracy, test–retest reproducibility, external validation, predictive performance, sensitivity, specificity, and clinical utility were not evaluated.

Several limitations should be acknowledged. First, the cross-sectional design precludes causal inference. In addition, disease duration differed substantially between the long COVID and ME/CFS groups, and disease-specific and disease-phase-related effects could not be fully separated. Complete-case requirements for MRI, clinical, and immunological data may also have introduced selection bias. Second, multiple correlation analyses were performed across numerous subfields and biomarkers, and no volumetric or correlation finding survived correction for multiple testing. Third, symptom assessment relied mainly on PS and did not include multidimensional fatigue assessments or detailed measures of post-exertional malaise, dysautonomia, cognitive dysfunction, psychiatric symptoms, or olfactory dysfunction. Fourth, the sample size was modest, and external validation is required. Fifth, mechanistic intermediates, such as circulating T follicular helper cell subsets, detailed PB and Treg phenotyping, and functional autoantibody assays, were not directly assessed. Finally, although FreeSurfer-based amygdala subfield segmentation is standardized and useful, volumetric assessment of small nuclei has inherent limitations; future studies using higher-resolution MRI, multispectral segmentation, or additional manual quality control are warranted (21).

In conclusion, although no finding survived correction for multiple testing, the exploratory results suggest that amygdala-related structural variation may be relevant to both long COVID and ME/CFS. The nominal, uncorrected within-group patterns centered on olfactory-related amygdala nuclei and GPCR autoantibodies in long COVID, and on amygdala structure, functional severity, and plasmablasts in ME/CFS; however, these patterns do not support a conclusion of between-group differences. These observations are hypothesis-generating and do not establish disease-specific mechanisms. Prespecified replication in larger longitudinal and independent cohorts is required.

## Data Availability

The datasets generated and/or analyzed during the current study are not publicly available because of participant privacy and ethical restrictions. Data may be made available by the corresponding author upon reasonable request and subject to institutional approval.

## Footnotes

1
http://surfer.nmr.mgh.harvard.edu/
